# Categorization of mHealth Coaching Technologies for Children or Adolescents With Type 1 Diabetes: Systematic Review

**DOI:** 10.2196/50370

**Published:** 2024-10-10

**Authors:** Pavel Trnka, Tahmineh Aldaghi, Jan Muzik

**Affiliations:** 1 Department of Information and Communication Technologies in Medicine, Faculty of Biomedical Engineering, Czech Technical University, Studničkova 7, Prague, 12800, Czech Republic, 420 777568945; 2 The Spin-off Companies and Research Results Commercialization Center, First Faculty of Medicine, Charles University, Prague, Czech Republic

**Keywords:** type 1 diabetes, adolescents, children, parents, mHealth, information technology, PRISMA

## Abstract

**Background:**

Managing type 1 diabetes in children and adolescents can be difficult for parents, health care professionals, and even patients. However, over the last decades, the quality of services provided to patients with diabetes has increased due to advances in IT.

**Objective:**

This study aims to comprehensively document the range of IT tools used in the management of diabetes among children and adolescents, with a focus on identifying the technologies most commonly used based on their frequency. In addition, the study aims to explore relevant methodologies for developing diabetes technology and provide valuable information to developers by delineating essential phases of the design process.

**Methods:**

The literature search was focused on MEDLINE (PubMed), Web of Science, and Google Scholar for relevant studies. Keywords such as “type 1 diabetes,” “adolescents,” “kids,” “mHealth,” “children,” and “coaching” were combined using Boolean operators. The inclusion criteria were open access, English-language papers published between 2012 and 2023 focusing on patients younger than 18 years and aligned with our research goal. The exclusion criteria included irrelevant topics and papers older than 18 years. By applying the PRISMA (Preferred Reporting Items for Systematic Reviews and Meta-Analyses) method, 2080 studies were recognized, and after selection, 33 papers were agreed upon between the researchers.

**Results:**

Four primary categories were defined: types of IT, methodology identification, purpose identification, and feature determination. Among these, mobile health (mHealth) apps emerged as the predominant type of information, garnering 27 mentions. In particular, user-centered design was identified as the most prevalent methodology, cited 22 times. The primary purpose of self-monitoring blood glucose values was mentioned 20 times, while patient education was the highest among common characteristics, with 23 mentions.

**Conclusions:**

Based on our research, we advocate for developers to focus on creating an mHealth app that integrates gamification techniques to develop innovative diabetes management solutions. This app should include vital functionalities such as blood glucose monitoring, strategies to improve hemoglobin A_1c_ levels, carbohydrate tracking, and comprehensive educational materials for patients and caregivers. By prioritizing these features, developers can enhance the usability and effectiveness of the technology, thereby better supporting children or adolescents with diabetes in their daily management endeavors.

## Introduction

### Background

A chronic condition of glucose metabolism known as “diabetes mellitus” is described by impaired insulin production and action. According to the etiopathology of diabetes, the 3 most common clinical classes are distinguished: type 1 diabetes (T1D), type 2 diabetes, and gestational diabetes mellitus [1,2]. T1D is the most common type of diabetes affecting children or adolescents, although it can occur in all age groups [3-6].

T1D is typically diagnosed at a young age. After a child is diagnosed with diabetes, the child and his caregiver or parents need a period of adaptation to the new situation. In addition, children or adolescents may struggle emotionally with their health conditions, especially those that are diagnosed early. Therefore, they need the support of their caregivers to manage their disease [6-8]. Caregivers are crucial supports for the ability of children or adolescents to self-manage T1D [6,7,9]. However, caregivers often report frustration, stress, and worry about their role [10]. This period after diagnosis can be challenging for both children and parents [8].

T1D requires a lifetime of self-management at home and in the community, which requires regular consultations with the health care team [11]. Intensive self-management is characterized by frequent self-monitoring of blood glucose (SMBG), physical activity, carbohydrate intake, and insulin doses by using multiple daily insulin injections or an insulin pump for basal and bolus insulin delivery [11,12]. Improved blood glucose control has been shown to reduce mortality and the incidence of serious and costly complications such as kidney and cardiovascular disease [11]. Achieving ideal blood glucose levels requires intensive self-management, which can be a challenge for young people [4]. Supporting adolescents and children in the self-management of T1D is an integral goal of health care. Health care professionals (HCPs) must guide insulin management and seek insight into the lived experiences of patients, such as social life, work, and school, to identify issues that influence self-management. In addition, adolescents or children with T1D often skip clinical visits, show a lack of adherence to monitor their hemoglobin A_1C_ (HbA_1c_) trends, and endanger their current and future health [13-15].

The quality of services provided to patients with diabetes has increased due to advances in IT over the past few decades [16]. Numerous studies have investigated the efficiency of using websites, apps, and messaging systems as tools of IT to manage diabetes [4-7,13,17-23]. In this regard, along with various types of information technologies, the number of diabetes apps available for download on the iOS App Store and Google Play to improve diabetes self-management has proliferated recently [5,7,24]. The mobile health (mHealth) apps improve patient education and communication with HCPs and peers conveniently and interactively [13,25], especially among children or adolescents, an age group that easily adopts new technology [26].

### Objective

**Figure 1.**
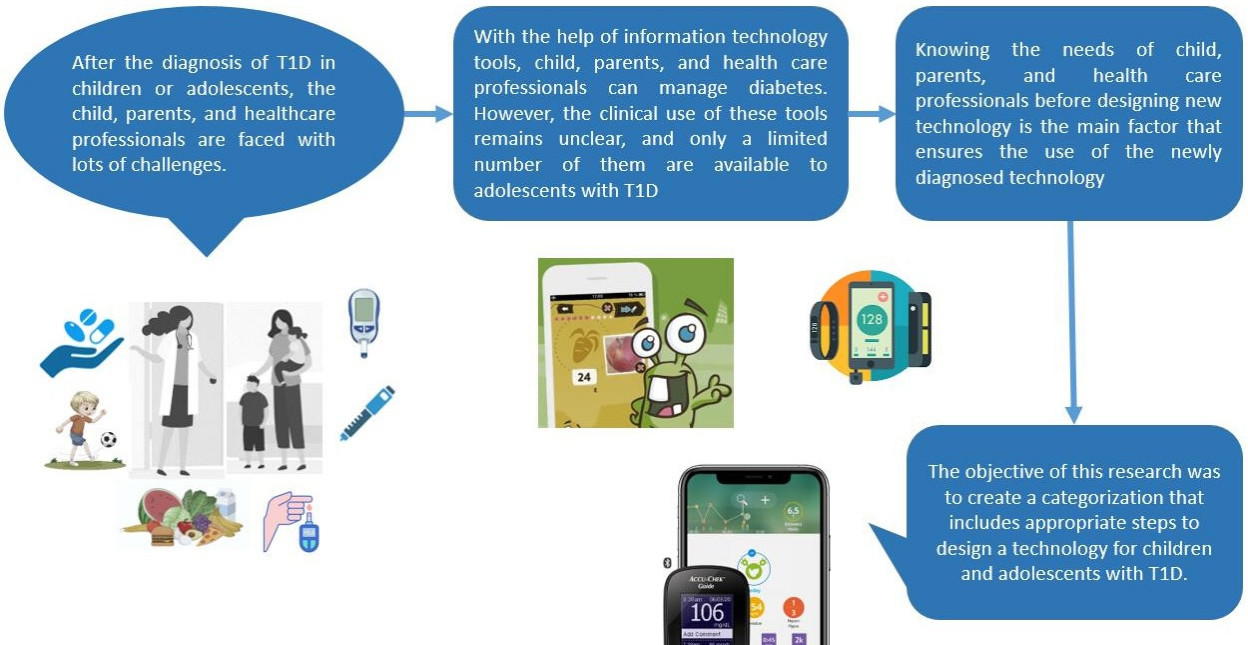
Graphical abstract. T1D: type 1 diabetes.

This study aims to thoroughly catalog the variety of IT tools used in the management of diabetes among children or adolescents. The main goal is to identify the technologies that are most widely used by noting their frequency. The study also intends to investigate several approaches that are relevant to the creation of diabetes technology. It plans to provide developers in this industry with useful information by outlining the fundamental phases involved in the design process. Figure 1 shows the graphical abstract of the paper.

## Methods

The electronic databases used for the search were MEDLINE (PubMed), Web of Science, and Google Scholar. Keywords were “type 1 diabetes,” “adolescents,” “kids,” “mHealth,” “children,” and “coaching.” These were combined using the Boolean operator “AND” and included their respective synonyms with the operator “OR.” The inclusion criteria for the studies included open access, papers published in English from 2012 to 2023, patients with diabetes younger than 18 years, and consistent with the research goal. The criteria for exclusion were irrelevance to the major topic, older than 18 years, conference papers, reports, and theses. Our systematic database search identified 2080 studies from 3 databases. The study selection is using PRISMA (Preferred Reporting Items for Systematic Reviews and Meta-Analyses) guidelines [27].

## Results

### Overview

**Figure 2.**
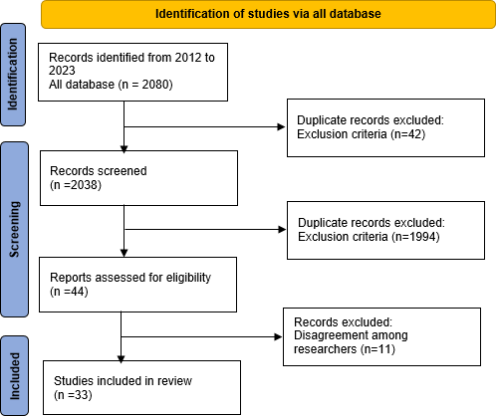
PRISMA (Preferred Reporting Items for Systematic Reviews and Meta-Analyses) flowchart.

Based on Figure 2, after filtering out publications based on duplication and exclusion criteria, only 44 remained. However, 11 of these were later excluded due to discrepancies among researchers. These papers did not achieve the consensus of researchers regarding their alignment with the objectives of the current research, leading to their exclusion. At the end of the selection process, 33 researchers fully agreed upon the papers. Of 33 research papers, 30 were in Google Scholar, 3 in PubMed, and nothing on Web of Science (after removing duplicates). Through exploration of the papers, it was initially found that the 3 main information technologies applied to the management of T1D among children or adolescents are the mHelath apps, the website, and the messaging system. Then, it became clear that the gamification, user-centered design (UCD), and text message systems were the 3 most popular methodologies to design new technology for diabetes. Finally, the procedures for creating a new technology for children or adolescents are as follows:

1. Types of IT
2. Methodological identification
3. Purpose identification
4. Feature determination

### Types of IT

Through reviewing the papers, it becomes apparent that there are 3 main types of IT that are used in the management of T1D among children or adolescents, which are shown in Table 1.

**Table 1.** Types of IT.

Platforms	References
mHealth^a^	[4,5,7,8,11-13,15-23,28-37]
Websites	[6,16,17,35,38,39]
Messaging system	[21,40,41]

a mHealth: mobile health.

### Methodology Identification

Three methods were recognized for designing an IT for the management of T1D in children or adolescents, which are summarized in Table 2.

A comprehensive overview of various information technologies and their respective features has been compiled in Table S1 in Multimedia Appendix 1 to maintain the study’s brevity and avoid unnecessary complexity resulting from an excess of tables.

**Table 2.** Methodology identification.

Methods	References
Gamification	[6,8,22,23,32,37,38]
Text message system	[21,40,41]
UCD^a^	[4,5,7,8,11-13,15-21,28-31,33-36,39]

a UCD: user-centered design.

### Purpose Identification

Upon reading the papers, it was concluded that recognizing the goal is a significant step in the development of a new technology. Table 3 presents the categorization of the goals extracted from the papers.

**Table 3.** Purpose identification.

Goal	References
Self-monitor blood glucose value	[4,6-8,11,12,16,17,22,28,29,32,33,35,37,38]
Improve HbA_1c^a^_	[5,7,12,20,29-31,36,40]
Self-manage insulin adjustment	[6,18,22,23,28,38]
Improve the life quality	[6-8,12,15,38]
Improve the communication among parents, adolescents or children, and HCPs^b^	[4,7,11,12,32]
Promote parents’ knowledge about diabetes	[16,17,32-34,36,42]
Enhance adolescents’ or children’s knowledge about diabetes	[6,7,12,18,19,21-23,30,33,38,41]
Track physical activity	[6,35,38]
Track food intake	[6,18,21,30,38]
Develop a device-agnostic cloud platform to host diabetes device data	[39]

a HbA_1c_: hemoglobin A_1c_.

b HCPs: health care professionals.

### Feature Determination

Identification of stakeholders is necessary before determining the characteristics of the technology. Identified stakeholders are parents, caregivers, HCPs, and children or adolescents with diabetes. In Table S1 in Multimedia Appendix 2, the lists of questions asked of stakeholders are attached. In the following, the summaries of the questions are mentioned.

Parents were often asked the following questions:

*What information should be included in the app; Do you like to see trends in a child’s blood glucose, HbA_1c_, and fasting blood glucose; How do you manage an adolescent’s diabetes; What challenges come with having children with diabetes; How much could a child with diabetes benefit from an application made specifically for this purpose.* [8,12,18,42]

Furthermore, the questions about designing technology with the intention of gamification were as follows:

*What is the most appropriate game design for a child with diabetes (taking care of a character, quizzes, runner game, storytelling); What is the most appropriate reward style that should be used in the game; What features would you like to have in the game.* [37]

Furthermore, questions such as, “If your HbA_1c_ is below the target range, what kind of information or message would you like to receive from the app?”; “What types of goals would you like to be able to set for yourself?”; “What was your experience and feelings about being diagnosed with diabetes?”; and “How do you deal with diabetes?” were frequently asked of adolescents [12,19]. Furthermore, “What thoughts and wishes are there regarding a virtual platform for children/adolescents?” [23]; this is a kind of question that was asked of HCPs.

Through the analysis of the responses of stakeholders to the questions, technological features emerge. Concise summaries of the features of the diabetes app are shown in Table 4.

**Table 4.** Feature determination.

Features	References
Reminders	[6,7,12,13,15,17,20,23,28,29,32-34,36-38,40]
Blood glucose tracking	[4,6-8,11,12,16,17,28,29,32,33,35-40]
Insulin administration	[5-8,12,17-19,23,28,29,31,32,36,38,40]
Carbohydrate counting	[5,7,8,12,13,15,17-21,23,29,30,32-34,37,39]
Patients’ education	[4,6-8,11-13,15,17-21,28,30,32-34,37,38,40,41]
Communication among stakeholders	[5,8,13,15,20-22,28,30,32,35,36,40]
Track physical activity	[6,8,16,17,19,23,28,32,33,35,38,39]
Parental involvement	[4,7,11,12,29,33,34]
Chat with peers	[8,13,15,20]

### Review of Current Technologies

Another point to consider when designing new technology is how successful the previously introduced technologies have been in achieving their goals. For the treatment of T1D in adolescents, Cafazzo et al [11] designed the Bant app using gamification incentives. They showed that the app improves the frequency of blood glucose monitoring in adolescents. Alsalman et al [23] proposed a gamification app in 2021; however, they have not provided any evidence of the app’s usefulness. In addition, Schmidt et al [22] created the gamification app in 2022, but they have not yet shared any user evaluation data. In 2017, den Akker et al [38] described a platform, PERMAGON, that integrates gaming and coaching for adolescents with type I diabetes. The purpose of the platform was to support patients and their caregivers in self-management of diabetes through educational games, monitoring, and motivational feedback. In 2018, Klaassen et al [6] conducted a study to evaluate the efficacy of PERGAMON. They asked participants: “Do you expect that the PERGAMON platform will support you in your diabetes management?” Approximately 33.33% (33/100) of the participants answered “yes,” which proved the effectiveness of the platform.

In 2017, Goyal et al [4] conducted a study on the Bant app to determine its impacts on HbA_1c_ and SMBG. They showed that the app has a positive impact on the use of SMBG data and glycemic control among youth. Klee et al [5] demonstrated that the Webdia app reduced HbA_1c_ levels in children who used it without increasing their prevalence of hypoglycemia. In 2017, Holtz et al [12] designed the MyT1DHero app using patient-centered methods to recognize their requirements. The participants confirmed that the new app would improve communication between parents and adolescents. The effectiveness of the MyT1DHero app in enhancing HbA_1c_ levels, adherence to diabetes care adherence, and quality of life was investigated in a new study by Holtz et al [7] in 2021. They showed that the app facilitates parent-adolescent communication, adherence to self-care, and improvement in quality of life. An Arabic-language application called Ana Alsukary was created in Saudi Arabia by Bitar et al [8]. This app consists of several features, one of which the users were interested in, namely, showing the location of stores that offer diabetes-friendly products. Caregivers of children with diabetes indicated that the app helps children understand their diabetes and adjust their lifestyle accordingly.

In 2017, Castensøe-Seidenfaden et al [13] designed a new app, Young with Diabetes (YWD), to support young people in the self-management of T1D. In 2018, Husted et al [15] examined the influence of YWD on young people’s self-management for 12 months. Participants mentioned that peer support reduced feelings of loneliness and helped them acquire knowledge and skills to manage T1D. Another study was carried out by Castensøe-Seidenfaden et al. in 2018 [20] to investigate the effectiveness of YWD in improving HbA_1c_ levels. The results demonstrated that the value of the app could not improve HbA_1c_ but could be a useful addition to self-management. In another study by Ledderer et al [28], adolescents using the Diapplo app appreciated the design and user interface of the app as well as the functions to have an overview of blood glucose values. However, they stated that the content of the app only partially met their needs. They suggested integrating social networks into the app and providing more knowledge on how to deal with chronic diseases. Albanese-O’Neill et al [17] created an online DSMES website and a separate mobile subdomain for fathers, Mobile Diabetes Advice for Dads (mDAD). They evaluated a prototype of the site with fathers’ participants. Information on mDAD was believed to be helpful by 55% (55/100) of fathers. Since many young adults with T1D struggle with the complex daily demands of adherence to their medical regimen and do not achieve glycemic control in the target range glycemic control, Stanger et al [36] provided a mobile app, SweetGoals, to study its ability to help young adults comply with their medical regimen. The results of their experiment will be published by December 2025.

Berndt et al [16] investigated the impact of Mobile Diab, a new mobile, website, and communication technology, to help the treatment and treatment of adolescents with T1D. They observed an improvement in the mean HbA_1c_ level. Frøisland et al [21] compared 2 types of technology, the Diamob application and diabetes message system, to determine which could have a better impact on diabetes management than others. The objectives of the Diamob app were communication between the patient and the health care team about carbohydrate consumption, insulin dosage, and types of physical activity. The diabetes message system has been used to send educational messages to patients and messages to HCPs when faced with obstacles in daily life. As a result, adolescents found both the Diamob and the diabetes message system practical as support for their diabetes self-management. They appreciated Diamob more than the text message app. In 2015, Frøisland and Årsand [30] developed and tested a new feature for Diamob that allowed one to take pictures of the food and then measure the carbohydrates in the food, the insulin dose needed, and the type and amount of physical activity. They concluded that adding this feature would go a long way toward helping young people understand the basics of diabetes. Hilliard et al [34] created and tested a new mHealth app for parents of teens with T1D, which is Type 1 Doing Well. Parents claimed that the app had high acceptance and the greatest suitability for providing information. The CanDIT (Canadian Diabetes Incentives and Technology) app was developed by Krmpotic et al [29]. The app was evaluated by a small number of youths. They stated that they use the app most often for SMBG. They reported that they liked the ability to set reminders and view their glucose history. They listed several suggestions for improving the app, such as the ability to personalize target blood glucose ranges, calculate insulin doses, enter blood glucose values, and track carbohydrate intake. Shetty et al [31] claimed that enabling young people with T1D to control their blood glucose levels during exercise is a complex challenge for HCPs. Therefore, they asked 10 young people with T1D to use the acT1ve mHealth app to help them exercise in real time. Participants indicated that the app improved their knowledge and boosted their confidence when exercising.

Zholdas et al [35] presented an information on technology–based mHealth monitoring system, including sensors, medical bracelets, and mobile devices with applications to calculate the probable change in a patient’s blood glucose level after the end of physical activity. After a test, they demonstrated a drop in blood glucose levels after physical activity, which confirms their hypothesis that there is a link between physical activity and blood glucose levels. To host data from diabetic devices and develop an ecosystem of software innovation to treat T1D care, Neinstein et al [39] created a device-agnostic cloud platform, Tidepool, to host data from diabetic devices and catalyze an ecosystem of software innovation for T1D management. They concluded that the Tidepool platform can solve 2 current problems in the T1D device landscape: limited access to T1D device data and poor interoperability of data from different devices.

Text messaging is another type of platform used by researchers. Bin-Abbas et al [40] conducted a mobile phone messaging service for children or adolescents with T1D. Three types of mobile phone messages have been sent to children through their parents, including informational messages, interactive messages, and multimedia messages. The results showed that HbA_1c_ levels were significantly reduced, parents’ knowledge of diabetes improved significantly, and text messages on mobile phones were a useful way of contacting each other between clinic visits. Zhang et al [41] conducted another study with the hypothesis that higher levels of participation in the intervention would yield better diabetes management and examined whether caregiver participation or other demographic factors were associated with the level of participation. They concluded that there was a good level of participation in a text messaging intervention for adolescents with T1D, and high participation was related to a greater improvement in caregiver-reported adherence. Race, ethnicity, and sex were the only demographic factors significantly related to the level of participation.

## Discussion

### Principal Findings

Management of diabetes can be difficult for children or adolescents, parents, and HCPs. To address this, technology plays a vital role in providing support. These modern technologies are characterized by significantly better cost-effectiveness, which is a stark contrast to traditional, more expensive alternatives. Unlike traditional systems, they eliminate typical total cost of ownership issues [43]. To better assist adolescents or children dealing with T1D, this study conducted a comprehensive assessment aimed at initially cataloging the range of IT tools used in diabetes management and pinpointing the most prevalent technologies used. Furthermore, the study delved into various methodologies relevant to the development of diabetes technology. Finally, it aims to provide developers in this field with valuable insights by delineating the fundamental phases inherent in the design process. Through a review of the literature, it became apparent that there are 4 key steps involved in designing a novel technology for the management of diabetes.

The initial step, as previously discussed, entails identifying the predominant IT used. In this regard, the mHealth apps emerged as the leader with 27 mentions, followed by websites with 5 mentions and text message systems with 3 mentions. This high frequency of mHealth apps is because children or adolescents have easy access to mobile phones, encouraging technology innovators to create more mHealth apps specifically for the management of diabetes. This finding corroborates previous research [13,25] indicating that mHealth apps effectively enhance patient education and communication with HCPs and children or adolescents conveniently and interactively.

The second step was to identify the main methodology for designing a new technology for children or adolescents with diabetes. It was evident that the most popular methodology is UCD, with 22 mentions. Gamification was referenced 8 times after UCD and messaging systems were cited 3 times. Gamification is the application of concepts of game design to the creation of settings that can encourage individuals to make healthier decisions [37]. Text message system is technique that uses a mobile phone to send messages to patients and their parents. UCD involved designing the app with the end user in mind, focusing on their needs, preferences, and behaviors throughout the design process. The age range of children or adolescents indicates that gamification may be the most successful strategy to motivate patients with diabetes to actively interact with the program, although UCD emerged as the most popular methodology. This can greatly support their attempts at self-management and encourage regular use, which will improve the management of their diabetes. Visualization plays a key role in captivating the interest of children and adolescents, making gamification an ideal avenue to incorporate various visually stimulating features. By creating a vibrant virtual world replete with colorful imagery, captivating stories, and charming characters, gamification provides the necessary motivation for patients with diabetes to use the app regularly. Through this engaging approach, the primary objective of helping patients manage their condition can be achieved in a lighthearted and enjoyable manner, particularly appealing to the younger demographic.

The third step involved identifying the core objectives that underlie the development of a new technology. Upon careful review of the literature, it became evident that SMBG values emerged as the primary purpose, cited 20 times. Following closely, the secondary objective was to improve HbA_1c_ levels, as mentioned 12 times. Finally, the objective of improving overall quality of life and increasing adolescents and children’s knowledge about diabetes was highlighted 9 times. Among other goals, the frequency of SMBG highlights how crucial it is to build new technologies with the primary goal of helping patients monitor their blood glucose levels effectively. It is imperative to give priority to features that optimize blood glucose monitoring since they help patients reduce the risk of hypoglycemia and other related problems, improving their overall health.

The final step in technology design involves careful consideration of its features. Upon review, it became evident that patient education ranked highest with 23 mentions, followed closely by carbohydrate counting with 22 mentions and blood glucose tracking with 21 mentions. This underscores the importance of incorporating these key features into the design of a technology. It is clear that for children and adolescents, independent learning can be challenging, emphasizing the necessity of providing intuitive icons to support patients and caregivers in managing diabetes effectively. In addition, accurate carbohydrate counting is paramount, as any miscalculation can lead to incorrect insulin dosing, posing significant risks to the patient’s well-being.

Based on our findings, we recommend that developers prioritize the creation of an mHealth app using a gamification methodology to design new diabetes management technologies. This approach should encompass essential characteristics such as blood glucose management, suggestions for improving HbA_1c_, carbohydrate counting, and comprehensive educational resources for both patients and caregivers. By emphasizing these aspects, developers can ensure the effectiveness and user-friendliness of the technology, ultimately enhancing its use in supporting children or adolescents with diabetes in their daily management routines.

### Limitations

The primary challenges encountered during this review stemmed from variations in the number of articles reviewed compared with the technologies identified. For instance, each group of papers (eg, [4,11], [7,12], [6,38], [21,30], and [13,15,20]) referenced a unique single app. In addition, article [8] mentioned 5 distinct apps; article [17] cited 2 separate apps; and article [37] included 3 apps, which add further complexity to the analysis. These differences make reviewing and analyzing the results more difficult and time-consuming.

## Supplementary material

10.2196/50370 Multimedia Appendix 1 Explanation of all information technologies and their features.

10.2196/50370 Multimedia Appendix 2 Explanation of stakholeders' questions.

10.2196/50370 Checklist 1 PRISMA (Preferred Reporting Items for Systematic Reviews and Meta-Analyses) checklist.

## Abbreviations

CanDIT: Canadian Diabetes Incentives and Technology
HbA_1c_: hemoglobin A_1c_
HCP: health care professional
mDAD: Mobile Diabetes Advice for Dads
mHealth: mobile health
PRISMA: Preferred Reporting Items for Systematic Reviews and Meta-Analyses
SMBG: self-monitoring of blood glucose
T1D: type 1 diabetes
UCD: user-centered design
YWD: Young with Diabetes
