# Supplementary material for: Categorization of mHealth Coaching Technologies for Children or Adolescents With Type 1 Diabetes: Systematic Review
Source: JMIR Pediatr Parent. 2024 Oct 10;7:e50370. doi: 10.2196/50370 (PMC11486482; doi:10.2196/50370)
Supplement: Multimedia Appendix 1 [file pediatrics-v7-e50370-s001.docx]

Multimedia Appendix Table S1. Features of each app

| **Name of project** | **Type of platform** | **Features** |
| --- | --- | --- |
| *Bant* [4,11] | App (iOS) | Banter chat  Trend wizard  Electronic logbook  Personal health record  Automatic data transfer  Fast transactions that take seconds  Share results with parents (ad hoc information)  Help adolescents identify blood glucose trends  Promote cognitive processing related to identifying and correcting the trend  Integrating rewards and incentives into the system to sustain engagement with the tool |
| *Diapplo* [28] | App^*^ | GPS  Physical activity  Consultation function  Reminder for the time of consultation  Information and advice about diabetes  Overview of the daily blood glucose value  Insulin adjustment and bolus calculator training  Information and advice about diabetes and its impact on traveling |
| *Webdia* [5] | App (android, iOS) | Calculate insulin injections  Visualization of patient data  Communication between the user and medical team  A complete list of foods with their nutrients and carbohydrate content |
| *MyT1DHero* [7,12] | App (Android) | Separate login for parents  Usage of insulin and reminders  Earn points based on app usage  Timing of the testing of blood glucose  Diabetes care adherence, and quality of life  Educational component for adolescents with diabetes  Improve the communication between parents and adolescents  Enter favorite foods or restaurants to get the nutritional information |
| *Ana Alsukary* [8] | App (Android) | Communication between peers  Displaying information about diabetes  Entering/viewing the blood sugar level  Displaying grocery stores' names and locations  Displaying educational videos about diabetes  Entering/viewing the time of insulin injections  Calculating the AC1/cumulative blood sugar level  Registration/log in: child, parents, healthcare experts |
| *Sukry* [8] | App (Android) | Monitor blood sugar levels, meals, and physical activities  Generate daily, weekly, and monthly reports with graphs  Follow up with the doctor and/or health educator through the app |
| *Sukry Alatfal* [8] | App (Android) | Includes stories, movies, and games  The possibility of adding doctor appointments  Serve children with diabetes while they are in school  The app's users are children with diabetes, parents, and teachers |
| *Sukaryat* [8] | App (iOS) | Alert notification  Display glucose level |
| *Edarat Alsukary* [8] | App (Android) | Educational information |
| *Design a app* [23] | App^*^ | Gamification  Reminders  Home screen  The food screen  App login screen  shopping cart icon  The unique code screen  Physical activities screen  Welcome message screen  The insulin injection screen |
| *The PERGAMON platform* [6,38] | Website | **Gaming:**   - Main game: an adventure/puzzle game played over a long time. Points (knowledge, experience, and evolution points) are needed to continue the main game. - Minigames: Used to communicate certain educational objectives related to chronic conditions using game metaphors.   **Virtual coach:**   - "Knows" the personal objectives of each user as well as their achievements in the real world and the game world. - Evaluates data regarding physical activity and blood glucose levels from sensors. - Offers guidance in the form of reminders, notifications, and suggestions about certain actions and events.   **Types of Goal/Tasks in gamification platform**:   - "My Sugar": Measuring blood glucose, reinforcing the relationship between diet and glucose levels, education about hypo and hyperglycemia, or taking notes regarding insulin use - "Physical activities": related to diabetes and physical activity and sports, such as setting some steps as a daily goal or learning about how to prepare to engage in sports. - "Active life": Support the user to self-manage their diabetes in a more introspective way rather than overtly directing their activities. - "Website Activities": related to the use of the system and the website, such as creating a profile or installing applications on the smartphone. |
| *Young with Diabetes (YWD)* [13,15,20] | App (Android, iOS) | Tip packages  Chat with peers  Information section  Carbohydrate counting  Receive daily T1DM tips  Contact HCPs and write notes  Reminders for self-management tasks  Provides information for parents on how to support their teens  Providing information about multiple T1DM topics, such as obtaining a driver's license |
| *Mdad (Mobile Diabetes Advice for Dads)* [17] | App^*^ | Exercise and activity  Reminders via text or email  Managing diabetes at school  Nutrition and meal planning  Insulin therapy (injection and pump)  Availability via mobile phone and tablet  Insurance coverage and financial support  Social support and psychosocial aspects of care  Sick days and ketone monitoring and treatment  Glucose monitoring, glucose targets, and pattern management  Multimedia features include a combination of videos, images, and animations  Management of hyper- and hypoglycemia and emergency glucagon administration |
| *DSMES* [17] | Website | Research  Age-related Topics  Technology (glucose monitoring, insulin delivery, emerging technology, living with technology)  T1D 101 (typically sorted into basic skills, basic knowledge, support and connections, newly diagnosed diabetes, and diabetes definition) |
| *Design a new app* [18] | App^*^ | Calculate insulin intake  Information for emergencies  Easy access to carbohydrate calculation  Inform and educate people in their local  Easy access to relevant guides and information  Provide security, control, and requested materials for school teachers, parents, and their children's friends |
| *Phone message* [40] | Messaging system | Three types of mobile phone messages:   - Informational messages: The messages covered general diabetes care knowledge, including diabetes symptoms, signs, pathophysiology, diagnosis, and management, including insulin therapy, diet therapy, psychotherapy, and diabetes press news. The messages reminded patients about checking blood glucose and insulin intake. - Interactive messages: Weekly interactive messages were sent to obtain patient information. For example, insulin doses could be adjusted if blood glucose readings were sent to the management team; HbA_1c_ levels could be calculated if the diabetes care team were provided with the blood glucose readings; and body mass index could be measured if the patients provided their body weights and height. - Multimedia messages: multimedia messages (MMS) were sent at the parents' request. These included video clips about procedures related to diabetes care, such as glucometer usage, insulin injection technique, or insulin pump insertion. |
| *Text messaging* [41] | Messaging system | Text-messaging features:   - 1 weekly personalized Important Value message - Adolescents received 4-5 text messages/week over the 8-week - Mood Booster messages (jokes and inspirational quotes) for boys and girls (different items), sent 2-3 times/week |
| *Jerry the Bear, Pandabetic, GlucoZor World* [37] | App (Android, iOS) | Gamification  Include storyteller  Check the character's blood glucose level  Feed the character with a variety of food  Managing a character's insulin administration |
| *Diapets, Commander Gage* [37] | App (iOS) | Gamification  Reminder  Help overcome the fear of insulin needles  Carbs count by taking care of a character |
| *Diabetes control, Gluco Bear* [37] | App (Android) | Gamification  Provide education regarding the best nutrition for diabetic patients |
| *Design a new app* [19] | App^*^ | Help section  Food intake  Insulin dosage  Physical activity  Visual diabetes diary  Select an avatar for each character  Play the educational game to earn rewards to personalize the avatar |
| *Diamob* [21,30] | App^*^ | Visualization: See the coherence of treatment and communication through pictures; visually identify unhealthy food; capture and visualize adolescents' food intake.  Access: easy access to educational messages; the possibility of contacting the healthcare provider and receiving an immediate response. |
| *Web-based SMS system* [21] | Messaging system | Send educational messages  Send messages to patients when they face obstacles in everyday life |
| *Diabetes Journey* [22] | App^*^ | Gamification  Problem-solving video game  Conversion with a coach in the app |
| *Type 1 Doing Well* [34] | App^*^ | Text message library  Watch psychoeducation videos  Reminder information about diabetes strengths  App for parents of adolescents with T1D |
| *Canadian Diabetes Incentives and Technology (CanDIT)* [29] | App (iOS) | Identify trends  Administer insulin  Parental involvement  Positive reinforcement  Log blood glucose value  Get points for gift cards  Carbohydrates counting  Wirelessly connect to the glucometer  A reminder of checking blood glucose levels |
| *Design a new technology* [33] | App (Android) | Features from child's views:   - An educational component - Send messages via the app to their parent - Customizable test reminders and BG ranges - Incentivized through the use of points accumulated based on usage - A moderated forum for the adolescents and the parents (separate forums) to post and discuss questions and comments with peers   Features from parent's views:   - An educational component - Send messages via the app to their child - Customizable test reminders and BG ranges - Review their child's history of BG values, carbohydrate intake, and physical activity from their phone - A moderated forum for the adolescents and the parents (separate forums) to post and discuss questions and comments with peers |
| *Design a new app* [32] | App^*^ | **Reminders;**  **Information and education;**  **Communication:**   - Create connections - Take pictures of meals - Clinician communication and feedback for reminders - Ability to communicate results (glucose levels and insulin dosing) to the clinic - Ability to flag things that affect management: exercise, illness, treating highs/lows   **Technological requirements:**   - Insulin calculator - Closed loop technology - Bluetooth compatibility - Carbohydrate calculator - Connectivity to social media - Estimate the HbA_1c_ level between clinic visits - Connect to Fitbit to track and record exercise   **Gaming:**   - Diabetes management trivia - Have a leaderboard and earn points that unlock rewards - Gamification is linked to effective management to maintain engagement - Avatars could be displayed positively or negatively based on current control |
| *Design a new technology* [35] | Website and app^*^ | Data processing layer  Continuous Glucose Monitoring, Fitness Bracelet  **Application layer**:   - Web application: intended for a doctor and serves for analyzing and visualizing patient data. - ​​​Mobile application: Observe previous readings from sensors, enter information about nutrition, blood glucose levels, the nature of the physical activity, and treatment recommendations compiled by a doctor​​. |
| *AcT1ve* [31] | App^*^ | Carbohydrate advice  Hypoglycemia treatment  Pre-exercise and post-exercise insulin |
| *SweetGoals* [36] | App^*^ | Web health coaching  Reports about goals met  Sets a personal goal for next week  Seek daily goals like the minimum CGM or CMBG  Uploads weekly data from a CGM or glucometer |
| *Mobile Diab system* [16] | App (Android, iOS), website | **Web-Based:**   - Lead to better glucose control - Track patterns and trends in the diabetes management process - Suitable for self-care, home care, family physicians, clinics, and hospitals - Provide risk monitoring; with automated alarm messages to the care provider   **Mobile app:**   - Track blood pressure - Track physical activity - Track weight and body size - Show the trend of blood glucose |
| *Tidepool* [39] | Website | Overview of physical activity  Overview of blood glucose trends  Overview of carbohydrate intake  Medical data storage: stores and retrieves medical data.  User data storage: manages users and communications to and from the user databases.  Blip application: combines individual patient data from multiple devices into a single integrated display. |

^*^The type of operating system was not mentioned in a related article.
