# Supplementary material for: Categorization of mHealth Coaching Technologies for Children or Adolescents With Type 1 Diabetes: Systematic Review
Source: JMIR Pediatr Parent. 2024 Oct 10;7:e50370. doi: 10.2196/50370 (PMC11486482; doi:10.2196/50370)
Supplement: Multimedia Appendix 2 [file pediatrics-v7-e50370-s002.docx]

Appendix 2: Stakeholders questions^*^

| **Authors** | **Methods** | **Parents' questions** | **HCPs' questions** | **Adolescents'/children questions** |
| --- | --- | --- | --- | --- |
| Holtz et al. [12] | Interview | 1. Is your adolescent using an app for tracking diabetes? 2. What types of information would be useful to have in the app? 3. Would you like to see trends in your child's blood glucose? HbA_1c_? Fasting blood glucose? Any others? 4. What types of messages would be encouraging to you in times of stress? 5. What types of communication to encourage your child to track its schedule do you prefer (texting, phone calls, face-to-face)? 6. Do you want some advice on how to manage your relationship with your adolescent? 7. Do you or your child log their blood glucose numbers? 8. How does your child typically talk to you (parents) throughout the day (texting, phone calls, face-to-face)? 9. What type of conversations (positive, happy, nagging, stressful) do you have with your child? 10. What makes the conversations friendly, stern, or stressful? 11. How do you manage your adolescents' diabetes? | - | 1. If your number is below the targeting range, what kind of information or message would you like to get from the app? 2. What kinds of goals would you like to be able to set for yourself? 3. What would you like to get if you achieve your goals? 4. Will you briefly talk about a typical day managing your diabetes? 5. Do you or your parents log your blood glucose? 6. When do you normally log in? 7. Can you describe a typical conversation with your parent about diabetes? |
| Bitar et al. [8] | Questionnaire | 1. Demographic data: age, gender, marital status, the age of the kid with diabetes, the age of diagnosis of diabetes. 2. The difficulty of having children with diabetes 3. The difficulties of using an application/technology to overcome diabetes challenges 4. What features do you prefer? | - | - |
| Alsalman et al. [23] | Questionnaire- interview | 1. Demographic information of participants: gender, age, level of education, kinship relationship with the child, household income, employment status (full-time, part-time, retired). 2. Demographic information of the children with diabetes: gender, age, and when the child was diagnosed with diabetes. 3. How much would your child with diabetes benefit from an application that is designed to manage his/her condition? 4. What is the most appropriate design of the game for your child with diabetes (taking care of a character, quizzes, runner games (ex. Crash games), storytelling)? 5. What is the most appropriate reward style that should be used in the game? (Points, levels, leaderboards (ranking of the players in the game)) 6. What are the features that you would like to have in the game for your diabetic child? (Reminders and notifications, social interaction, friends' challenges) 7. What are the behaviors that you struggle to change in your child to manage his/her condition (physical activities, managing the medication, nutrition problems, monitoring the blood glucose level, mental issues)? 8. Suggest any other comment. | 1. What are your thoughts and wishes regarding a virtual platform for children/adolescents? | - |
| Albanese-O'Neill et al. [42] | Interview | 1. Demographic data of parents: age, child age, diabetes duration, race/ethnicity, education, household income, employment status (full-time, part-time, retired), single mother (father), or couple 2. Technology use: have a smartphone, use a smartphone (daily, text and message only, use apps, search for information), desktop computer, laptop computer, tablet 3. Willingness to use their smartphone to receive diabetes education (per week, per day) and numbers of minutes per week to learn about diabetes (minutes) 4. Diabetes educational needs: diabetes search, how to take care of a sick diabetes child, information about continuous glucose monitors, help for diabetes challenges, diabetes, and nutrition, diabetes, and exercise, calculating and adjusting insulin dose, managing ketones, information about insulin pumps, diabetes at school, hyperglycemia, hypoglycemia, how insulin works, why/how diabetes happens, carbohydrate counting, ordering diabetes supplies, self-monitoring of blood glucose, how to use a blood glucose meter, giving insulin injection | - | - |
| Andersen et al. [18] | Interview | 1. How do you manage a child with diabetes? | - | 1. How did you feel when you realized you had diabetes? 2. How did you deal with diabetes? |

^*^Table only includes those articles that mention information about asking questions from stakeholders.
